# Thermodynamic Evaluation and Sensitivity Analysis of a Novel Compressed Air Energy Storage System Incorporated with a Coal-Fired Power Plant

**DOI:** 10.3390/e22111316

**Published:** 2020-11-18

**Authors:** Peiyuan Pan, Meiyan Zhang, Weike Peng, Heng Chen, Gang Xu, Tong Liu

**Affiliations:** Beijing Key Laboratory of Emission Surveillance and Control for Thermal Power Generation, North China Electric Power University, Beijing 102206, China; ppy917@163.com (P.P.); zmyncepu@163.com (M.Z.); kenneth_pwk@163.com (W.P.); xgncepu@163.com (G.X.); liut@ncepu.edu.cn (T.L.)

**Keywords:** compressed air energy storage, coal-fired power plant, system integration, feedwater heating, thermodynamic analysis

## Abstract

A novel compressed air energy storage (CAES) system has been developed, which is innovatively integrated with a coal-fired power plant based on its feedwater heating system. In the hybrid design, the compression heat of the CAES system is transferred to the feedwater of the coal power plant, and the compressed air before the expanders is heated by the feedwater taken from the coal power plant. Furthermore, the exhaust air of the expanders is employed to warm partial feedwater of the coal power plant. Via the suggested integration, the thermal energy storage equipment for a regular CAES system can be eliminated and the performance of the CAES system can be improved. Based on a 350 MW supercritical coal power plant, the proposed concept was thermodynamically evaluated, and the results indicate that the round-trip efficiency and exergy efficiency of the new CAES system can reach 64.08% and 70.01%, respectively. Besides, a sensitivity analysis was conducted to examine the effects of ambient temperature, air storage pressure, expander inlet temperature, and coal power load on the performance of the CAES system. The above work proves that the novel design is efficient under various conditions, providing important insights into the development of CAES technology.

## 1. Introduction

The global energy demand is soaring and still mainly relies on fossil fuels, which has caused energy shortage and climate change, thereby it is necessary for the world’s energy policy to move rapidly towards renewable, efficient, and flexible energy systems [1]. In the last decade, enormous growth has occurred in renewable energy sectors around the world, particularly in Northern America, Western Europe, and China [2]. However, the exploitation of renewable energy sources may be limited by their uncertainties, probabilities, and fluctuating behaviors [3]. Fossil fuels can be utilized to offer energy following customers’ demands and they are readily storable when not required, nevertheless, some renewable energy sources (such as solar energy, wind energy, etc.) are supposed to be harvested when they are available and may be stored until they are needed [4]. The continuing increment of renewable energy will depend on whether renewable energy systems can provide high-value energy on demand, and energy storage techniques can transform intermittent renewables for this purpose [5]. To achieve a higher fraction of renewable electricity, adequate storage equipment is essential and allows immediate renewable resources to be captured and kept until they are required [6]. Hence, numerous researchers have been dedicated to energy storage technology due to its superiorities in reducing energy consumption and financial costs, and it is probably applied as an alternative energy source as well [7]. Plenty of the literature has demonstrated the importance of energy storage techniques. For both utilities and their customers, energy storage can bring multiple benefits: (1) improving the operation efficiency; (2) diminishing primary fuel consumption; (3) promoting the security of energy supply; and (4) reducing environmental influence [8]. As energy storage may contribute to stable renewable electricity for customers, it is predicted that the requirement for energy storage will become triple the present value by 2030 [9].

According to the energy forms of stored electricity, energy storage systems can be principally classified into five categories, including mechanical, electrochemical, chemical, electrical, and thermal [10]. Compared with other energy storage methods, the mechanical method, primarily involving flywheel energy storage, pumped hydro energy storage, and compressed air energy storage (CAES), possesses several advantages especially in terms of environmental impact, costs, and sustainability [11]. In recent years, CAES has attracted massive attention because it can provide longevity, minimum environmental influence, reliability and availability, and economic assets [12]. As reported in Reference [13], the Huntorf (Germany) and McIntosh (USA) CAES facilities started commercial operation a few decades ago and several additional CAES commercial projects are being planned or under construction. Within a CAES system, the charging process is accomplished by using electrically driven compressors to convert electricity into the potential energy of pressurized air, subsequently, the pressurized air is stored in storage vessels until it is requested to be released for producing electricity again via the expansion in expanders (EXPs) [14]. CAES is recognized as one promising option for the combining renewable energy sources-based plants with electricity supply, and it has a huge potential to compensate for the fluctuating drawback of renewable energy [15].

However, a variety of both technical and economic limitations still exist for the application of CAES, for instance, high capital costs and negatively affecting the profitability of the grid, relatively low cycle efficiencies, geological restrictions, and uncertainties, etc. [14]. To address these issues, much work has been devoted to the optimization of CAES cycles [16,17,18], the development of related equipment [19,20,21], and CAES operation strategies [22,23,24]. Besides, integrating CAES systems with other energy systems or power cycles is regarded as an effective approach to cover the shortages of CAES and improve its performance. Houssainy et al. [25] proposed a high-temperature CAES system that can eliminate the necessary combustion and emissions in conventional CAES plants, and the hybrid configuration combines two stages of heating through separate low-temperature and high-temperature thermal energy storage units. Alsagri et al. [26] incorporated a small-scale organic Rankine cycle (ORC) unit with the compression part of a CAES system to exploit the produced heat in compressing the airflow for providing a portion of the required work of the compressors, thereby promoting the cycle efficiency of the CAES system. To reduce energy dissipation, supply power and potable water, Razmi et al. [27] combined CAES with a multi-effect desalination system, wherein compression heat during charging is delivered to the desalination unit, and during discharging the residual energy of the turbine exhaust is reassigned to the desalination unit after passing through the recuperator. Wu et al. [28] developed a hybrid energy storage system based on the integration of thermochemical energy storage and CAES, which can store energy from wind, solar, and/or off-peak electricity simultaneously. Razmi et al. [29] designed an environmentally-friendly hybrid system involving absorption-recompression refrigeration, CAES, and wind turbines to serve retail buildings, where wind turbines are employed to yield electricity during off-peak hours, and a booster vapor compressor is utilized between the generator and condenser of the conventional absorption cycle to fulfill sufficient heat transfer between them. Bartela et al. [30] developed an energy storage system including the idea of energy storage using compressed air and the idea of energy storage using hydrogen, and the thermal integration of two sub-systems allows the efficient storage of a large quantity of energy based on the pressure tanks with limited volumes. Llamas et al. [31] put forward a CAES system integrated with an anaerobic digester, which use the generated heat to support the production and storage of biogas as chemical energy storage, and the latter will be exploited in the air expansion stage to maximize the performance of the overall process. Diyoke et al. [32] employed a CAES system and a biomass gasification system in a hybrid mode to synchronously produce electricity and warm water, and this hybrid system is designed to meet the peak load power demand of 1.3 MW for domestic use. Roushenas et al. [33] presented an integrated system combining solid oxide fuel cell with CAES and turbocharger for simultaneous production of hot water and power in retail buildings, and the solid oxide fuel cell subsystem is the primary power generation unit, coupled with the CAES and other supporting auxiliary cycles.

From the above literature review, it is clear that extensive research has been performed on the integration of CAES with other energy systems or power cycles. However, far less attention has been devoted to combining CAES with coal-fired power plants. A large-scale coal power plant is a complex thermodynamic cycle containing various energy conversion processes and multiple energy and substance inflows and outflows. Hence, there is tremendous potential in improving a CAES system by incorporating it with a coal power plant. Zhang et al. [34] integrated CAES with the regenerative subsystem of a coal-fired power plant and verified that the hybrid design is effective and feasible. Whereas, more work is necessary to be conducted on exploring the integration of CAES and coal power plants and the inherent mechanism of performance enhancement. Against this backdrop, a conceptual solution to integrate a CAES system with a coal power plant has been proposed in the current research. In the new scheme, the compressed air cooling and heating processes of the CAES system are entirely connected with the feedwater heating process of the coal power plant. Consequently, the cycle efficiency of the CAES system is enhanced and the thermal energy storage equipment for a conventional adiabatic CAES system is eliminated. Based on a 350 MW coal power plant, the thermal performance of the hybrid design was assessed. Furthermore, energy and exergy analyses were carried out to reveal the root cause of performance improvement. Finally, a sensitivity investigation was conducted to examine the performance of the hybrid system under various conditions. The findings of this study may provide some valuable guidance for the advancement of CAES technology.

## 2. System Description

**Figure 1:**
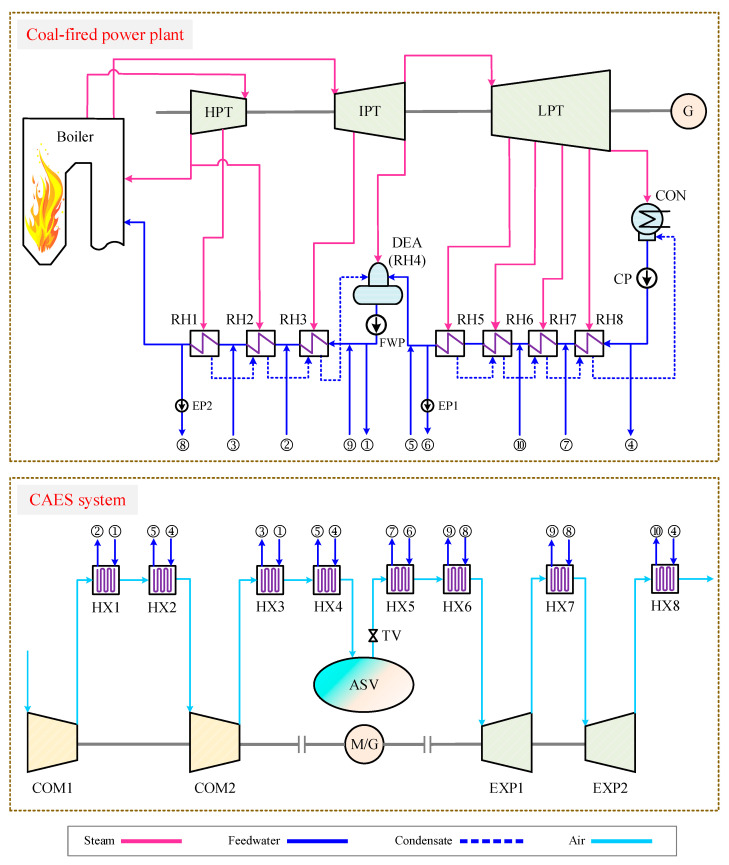
Diagram of the proposed compressed air energy storage (CAES) system incorporated with a coal-fired power plant.

This paper developed a conceptual CAES system organically integrated with a coal-fired power plant. As depicted in Figure 1, the connections between the air cooling & heating processes of the CAES system and the feedwater heating process of the coal power plant have been established based on eight heat exchangers (HXs). After the compression in Compressor 1 (COM1), the air is cooled by the HX1 and HX2, which use the feedwater fetched from the feedwater pump (FWP) outlet and condensate pump (CP) outlet as cooling mediums. Then the cooled air is forced into the COM2 and further compressed. Prior to the air storage vessel (ASV), the air transfers energy to the feedwater in the HX3 and HX4, and fed into the ASV with a low temperature. By finishing the compression process, the electric energy is converted into the compressed air energy and stored in the ASV. Furthermore, partial feedwater of the coal power plant is warmed by the air out of the COMs, which can save the extraction steam used for feedwater heating and conduce to reducing the fuel consumption of the coal power plant. During the discharging process of the CAES system, the high-pressure air is released from the ASV and heated by the HX5 and HX6 using the feedwater brought from the coal power plant. Afterwards, the air is exploited to drive the Expander 1 (EXP1) for power production. Previous to the EXP2, the air absorbs heat from the feedwater again, and then it is poured into the EXP2. When the air is discharged out of the EXP2, the waste heat of the air is recovered by the low-temperature feedwater in the HX8. Finally, the electricity generated by the CAES system’s generator (G) can be supplied to the grid and the round trip of the CAES system is accomplished. Through the integration with the coal power plant, the overall efficiency of the CAES system can be improved and more power will be produced from the stored air. Moreover, the thermal energy storage equipment of a regular CAES is unnecessary in the current design, which can dramatically diminish the capital costs of the CAES system.

## 3. System Simulation

### 3.1. Parameters of Reference Coal-Fired Power Plant

For the purpose of case study, a classic supercritical power plant has been selected. The power plant primarily involves a pulverized coal-fired boiler, extraction condensing steam turbine, generator, and feedwater heating system with eight regenerative heaters (RHs). This plant is real and serves in Northern China, and the actual operating parameters are close to its design data. The design data of the reference plant has been adopted for model simulation and performance evaluation in the following analysis, which was obtained from the owner of the plant. The design data was originally derived by the relevant manufacturers that built/provided devices for this plant, for instance, the boiler manufacturer and turbine manufacturer. Table 1 presents the basic data of the reference coal power plant. Once the coal of 42.29 kg/s is burned under the 100% load, 330.52 MW net power is produced with an energy efficiency of 41.69%. The feedwater is warmed from 32.6 to 276.4 °C by the extraction steam during the feedwater heating process, and the feedwater with various temperatures can probably be employed to cool or heat the compressed air of the CAES system.

### 3.2. Model Development and Simulation

Several modeling tools can be implemented for power system simulation, and a few software packages are available on the market, such as Aspen, APROS, HYSYS, MATLAB/Simulink, and EBSILON Professional [35]. In this paper, the EBSILON Professional (STEAG Energy Services GmbH, Germany) platform has been adopted to simulate the studied systems. EBSILON Professional is an “all in one” solution for power plant planning and development, which is suitable for all power plant types and other thermodynamic processes. Elements of modeling in the EBSILON Professional are the components and control elements, for both of which the specification values can be assigned internally or externally. A matrix solution process is adopted, which requires the linearization of all dependencies.

**Figure 2:**
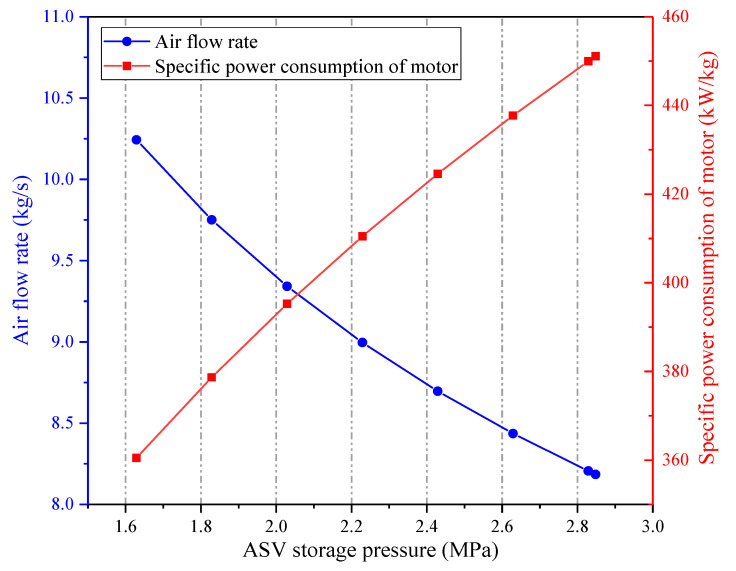
Divided periods of the charging process of the CAES system.

During the charging or discharging process of the CAES system, most of the parameters of the compressors/expanders and the parameters of the HXs can be maintained constant, however, the pressure of the ASV is variable during the charging process. With the rise of the ASV pressure during the charging period, the outlet pressure of the COM2 increases as well. As storage pressure of the “ASV” module in EBSILON Professional cannot change automatically, the storage pressure and the COM2 outlet pressure are adjusted manually by dividing the dynamic charging process into several steady processes [36], as displayed in Figure 2. The storage pressure and the COM2 outlet pressure are maintained constant in each tiny steady period.

**Figure A1:**
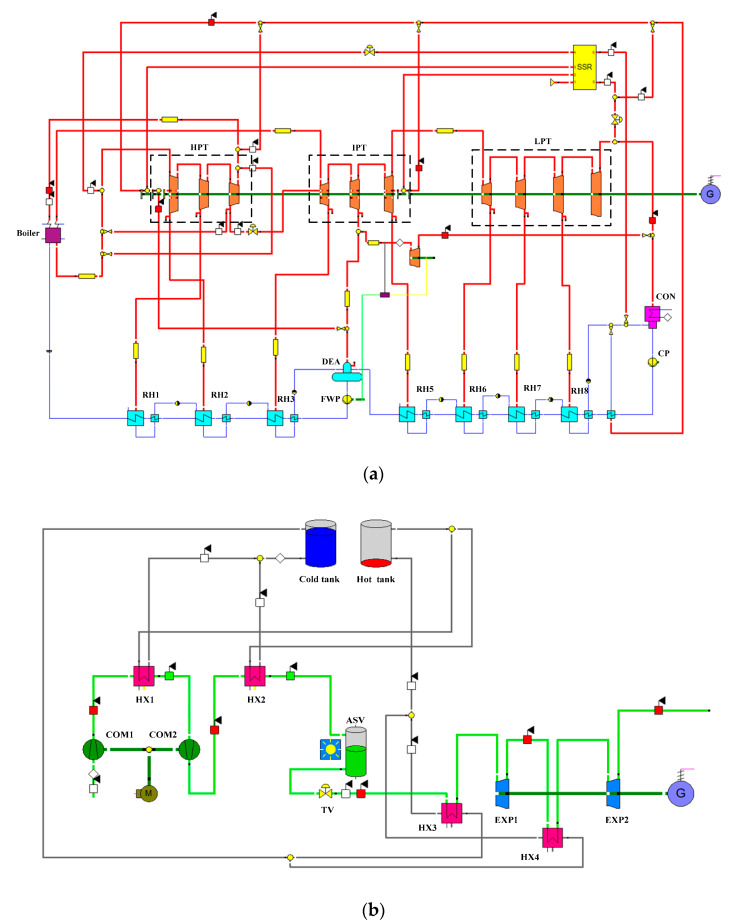

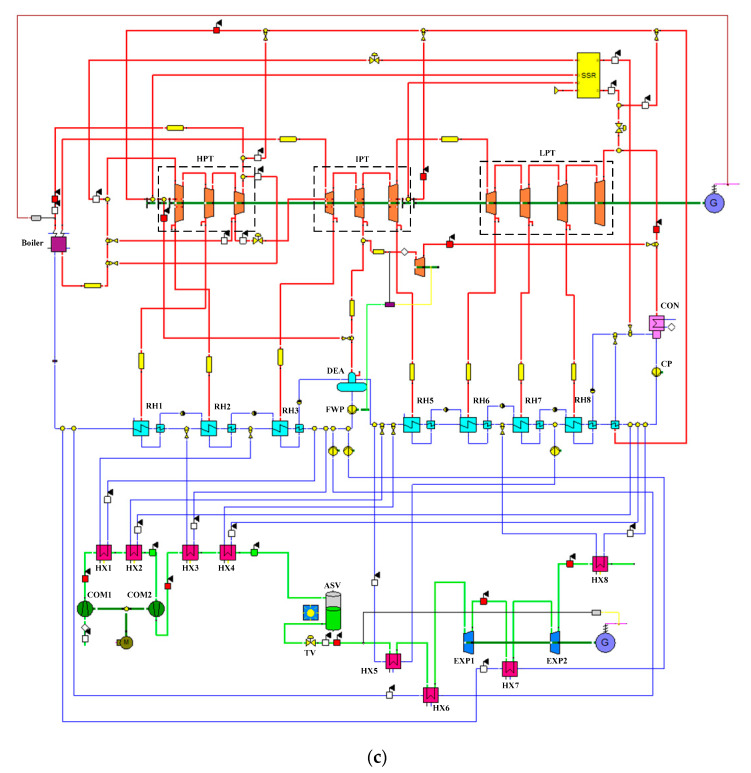
Simulation models of the studied systems, (**a**) reference coal-fired power plant, (**b**) reference CAES system, (**c**) proposed CAES system.

The simulation models of the studied systems (see Figure A1, displayed in Appendix A) were established on the EBSILON Professional platform using its inbuilt modules. The simulation models were validated by comparing the simulation results to the design data of the reference power plant and the CAES system in Ref. [37]. Table 2 and Table 3 indicate that the simulation results are quite close to the design values, thereby the simulation models are accurate and reliable.

## 4. Thermodynamic Analysis

### 4.1. Basic Hypotheses

As compared to the single coal power plant, the hybrid design organically combines the charging and discharging processes of the CAES system with the coal power production process. To assess the performance of the new CAES system, several assumptions are essential for the reference coal power plant and integrated system.

(a) The net power generated from coal is deemed as constant;
(b) The air of the CAES system is regarded as an ideal gas, which consists of 75.53% N_2_, 21.14% O_2_, 1.29% Ar, and 0.04% CO_2_ (mass fraction). The influence of the humidity in the air is neglected (The simulation results indicated that the outlet temperatures of COMs and EXPs vary less than 1 °C and the power consumption/generation changes less than 1% under the consideration of the humidity in the air);
(c) The environmental temperature and pressure are 25.0 °C and 101.325 kPa.
(d) The effect of the surroundings is not considered.

### 4.2. Parameters of Proposed System

Based on the concept to integrate the CAES system with the coal power plant, we examined several configurations and numerous specifications previously, and the most suitable configuration has been presented in the current paper. A few fundamental parameters of the hybrid system were assigned as boundary conditions to determine the maximum performance of the hybrid system, mainly based on the maximal round-trip efficiency. Many possibilities (e.g., number of HXs, HX temperature differences, HX inputs/outputs, etc.) were considered to optimize the hybrid design.

The basic parameters for the new CAES system were determined according to References [35,38,39], as listed in Table 4. The air storage temperature and pressure are set as 50.0 °C and 2.85 MPa, respectively. The requisite ASV is composed of several pressure tanks, and the total volume is calculated to be 17,940 m^3^. The isentropic efficiencies of the COM and EXP are chosen as 88%. A total of 8 h is spent to compress the air for storing energy when there is redundant electricity on the grid, and the stored energy will be used for power generation in 2 h.

**Figure 3:**
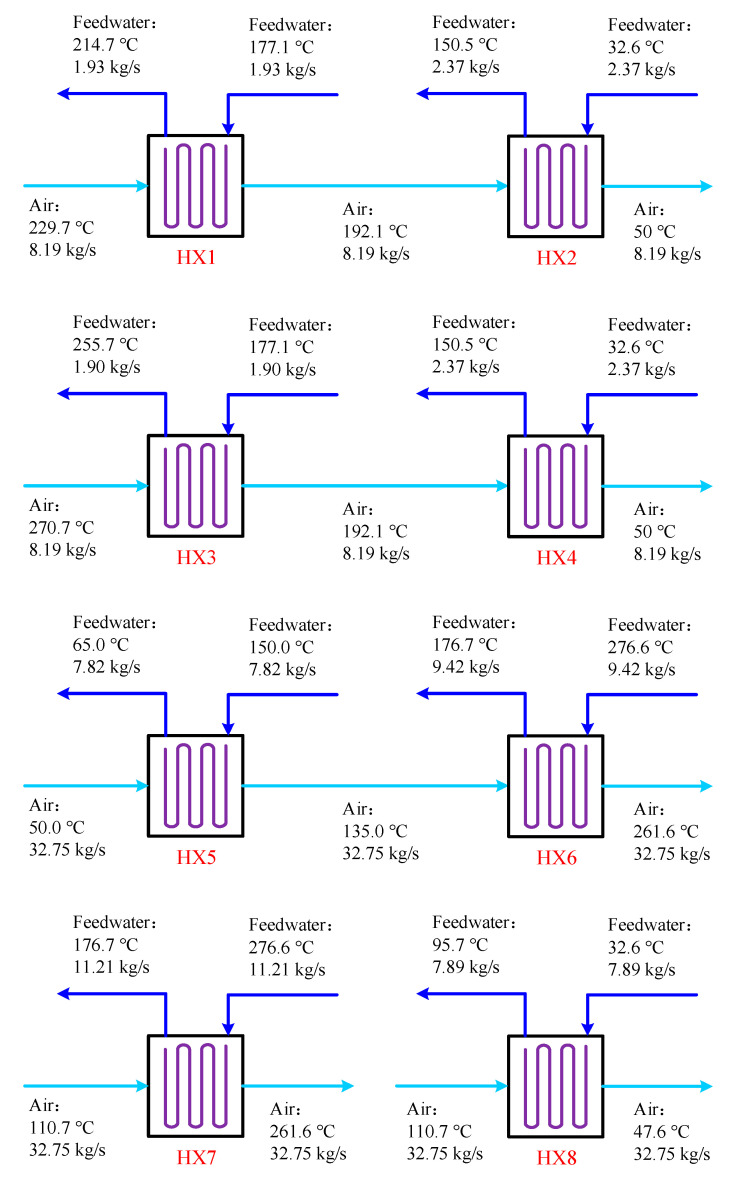
Main parameters of the heat exchanges (HXs) in the proposed CAES system.

The feedwater of the coal power plant is exploited to recover/supply heat energy from/to the compressed air in the HXs, and the parameters of the HXs are illustrated in Figure 3. As the HXs are employed to accomplish the heat exchange between air and water, they are designed to be tube-type heat exchangers. Spiral finned tubes are suggested to be used as the heating surface. The corrosion of air is not severe, thereby normal carbon steel can be applied as the material of the HXs. During the charging process, the air out of the COM1 passes though the HX1 and HX2, and conveys heat to the feedwater taken from the FWP outflow and the CP outflow. Subsequently, the feedwater is sent back to the RH2 inlet and the deaerator (DEA) inlet. HX3 and HX4 utilize the feedwater from the FWP outflow and the CP outflow for air cooling as well, but the heated feedwater is delivered to the RH1 inlet and the DEA inlet. After finishing the above process, 3.34 MW heat is recovered from the hot compressed air, and the high-pressure air of 50.0 °C is eventually fed into the ASV. While the compressed air is released from the ASV, the air is heated by feedwater fetched from the RH5 outflow and RH1 outflow previous to the EXP1. The air that has expanded in the EXP1 is reheated by the feedwater extracted from the RH5 outflow, and then enters into the EXP2. Besides, the air discharged from the EXP2 is cooled by the feedwater from the CP outflow and its waste heat can be recouped. During the discharging process, the heat of 12.12 MW is transferred from the feedwater to the air for promoting the air temperature, and the waste heat of 2.08 MW is recovered from the exhaust air.

### 4.3. Energy Analysis

During the charging and discharging processes of the CAES system, the net power output of the coal power section is maintained identical in the integrated scheme, which is achieved by regulating the fuel consumption of the coal power plant. However, the charging and discharging processes of the CAES system will affect the operation of the coal power plant. The installation of the CAES system is considered to benefit the flexibility of the power grid, but the flexibility of the coal power section is nearly fixed. Regarding the efficiency measurement, the system boundaries (CAES system, coal-fired power plant, and hybrid system) were separated by assuming that the coal-to-electricity efficiency and the coal power output are maintained constant (Equations (1) and (2)) for the single coal power plant and the hybrid power system. Thus, the coal amount used for power generation by the coal power plant is regarded as identical for the single coal power plant and the hybrid power system. Furthermore, the coal consumption variation caused by the integration is considered in the energy balance of the CAES system, formulated as Equations (3)–(5).

(1) $$P_{c,\mathrm{ref}}=P_{c,\mathrm{hyb}}$$

(2) $$\eta_{c-e,\mathrm{ref}}=\eta_{c-e,\mathrm{hyb}}$$

(3) $$W_{\mathrm{in},\mathrm{CAES}}=W_{\mathrm{out},\mathrm{CAES}}+\Delta Q_{\mathrm{CAES}}$$

(4) $$W_{\mathrm{in},\mathrm{CAES}}=P_{M,\mathrm{CAES}}\times t_{\mathrm{ch}}+\Delta m_{c,\mathrm{ch}}\times q_{c,\mathrm{net}}\times \eta_{c-e,\mathrm{ref}}\times t_{\mathrm{ch}}+\Delta m_{c,\mathrm{disch}}\times q_{c,\mathrm{net}}\times \eta_{c-e,\mathrm{ref}}\times t_{\mathrm{disch}}$$

(5) $$W_{\mathrm{out},\mathrm{CAES}}=P_{G,\mathrm{CAES}}\times t_{\mathrm{disch}}$$

where $P_{c,\mathrm{ref}}$ and $P_{c,\mathrm{hyb}}$ are the net powers generated from coal in the reference coal power plant and hybrid power system, kW; $\eta_{c-e,\mathrm{ref}}$ and $\eta_{c-e,\mathrm{hyb}}$ are the coal-to-electricity efficiency in the reference coal power plant and hybrid power system; $W_{\mathrm{in},\mathrm{CAES}}$ and $W_{\mathrm{out},\mathrm{CAES}}$ are the work input and work output of the CAES system, kWh; $\Delta Q_{\mathrm{CAES}}$ is the energy loss of the CAES system, kWh; $P_{M,\mathrm{CAES}}$ is the power consumption of the CAES system’s motor, kW, which is provided by the grid; $t_{\mathrm{ch}}$ and $t_{\mathrm{disch}}$ are the charging time and discharging time of the CAES system, h; $\Delta m_{c,\mathrm{ch}}$ and $\Delta m_{c,\mathrm{disch}}$ are the coal consumption variations due to the integration during the charging process and discharging process, kg/s; $q_{c,\mathrm{net}}$ is the net caloric value of the coal, kJ/kg; $P_{G,\mathrm{CAES}}$ is the power output of the CAES system’s generator, kW, which is sent to the grid.

The round-trip efficiency (RTE, %) is a widely used performance indicator for a CAES system [20], which is based on the energy balance of the CAES system. The round-trip efficiency is defined as the ratio of total power output to total power input. In this paper, the round-trip efficiency is formulated as Equation (6). The total power output is the power production of the CAES system’s generator, and the total power input includes the motor power consumption and the coal consumption variation that is converted into electricity.

(6) $$\mathrm{RTE}=\frac{W_{\mathrm{out},\mathrm{CAES}}}{W_{\mathrm{in},\mathrm{CAES}}}$$

Besides, the energy storage density (ESD, kJ/m^3^) has been defined as Equation (7), which indicates the ratio between the total energy output during the discharging process and the air storage vessel size.

(7) $$\mathrm{ESD}=\frac{W_{\mathrm{out},\mathrm{CAES}}}{V_{\mathrm{ASV}}}$$

where $V_{\mathrm{ASV}}$ is the volume of the ASV, m^3^.

Regarding the global performance of the hybrid system, the overall efficiency of the hybrid system ($\eta_{\mathrm{hyb}}$, %) is defined as Equation (8).

(8) $$\eta_{\mathrm{hyb}}=\frac{P_{G,\mathrm{CAES}}\times t_{\mathrm{disch}}+P_{c,\mathrm{hyb}}\times (t_{\mathrm{ch}}+t_{\mathrm{disch}})}{P_{M,\mathrm{CAES}}\times t_{\mathrm{ch}}+m_{c,\mathrm{ch}}\times q_{c,\mathrm{net}}\times t_{\mathrm{ch}}+m_{c,\mathrm{disch}}\times q_{c,\mathrm{net}}\times t_{\mathrm{disch}}}$$

Moreover, the total coal consumption variation in the charging and discharging process ($\Delta m_{c,\mathrm{tot}}$, kg) and the total coal power consumed by the CAES system ($W_{c,\mathrm{CAES}}$, kWh) were also considered, which are formulated as follows.

(9) $$\Delta m_{c,\mathrm{tot}}=\Delta m_{c,\mathrm{ch}}+\Delta m_{c,\mathrm{disch}}$$

(10) $$W_{c,\mathrm{CAES}}=\Delta m_{c,\mathrm{ch}}\times q_{c,\mathrm{net}}\times \eta_{c-e,\mathrm{ref}}\times t_{\mathrm{ch}}+\Delta m_{c,\mathrm{disch}}\times q_{c,\mathrm{net}}\times \eta_{c-e,\mathrm{ref}}\times t_{\mathrm{disch}}$$

The energy performance of the integrated scheme was assessed based on the reference coal power plant under its 100% load condition, as illustrated in Table 5. During the charging process of the CAES system, the surplus electricity of 3.69 MW is consumed by the CAES system’s motor to maintain the continual compression process for 8 h. The compressed air releases heat to the feedwater drawn from the coal power plant, which results in a decrement of 0.09 kg/s in the coal consumption rate, while the net power production of the coal power plant is invariable. During the discharging process, the coal consumption rate rises from 42.29 kg/s to 42.74 kg/s, as the feedwater is taken from the coal power plant to warm the air into the EXPs. In the charging period (8 h), the coal power of 5.41 MWh can be conserved and 7.08 MWh coal power is required by the CAES system during the discharging period (2 h). In sum, 1.67 MWh additional coal power is consumed by the CAES system in the whole process. The proposed CAES system can yield 20.00 MWh net power in the discharging process with an energy storage density of 4.01 MJ/m^3^, and the round-trip efficiency the CEAS system can reach 64.08%.

**Figure 4:**
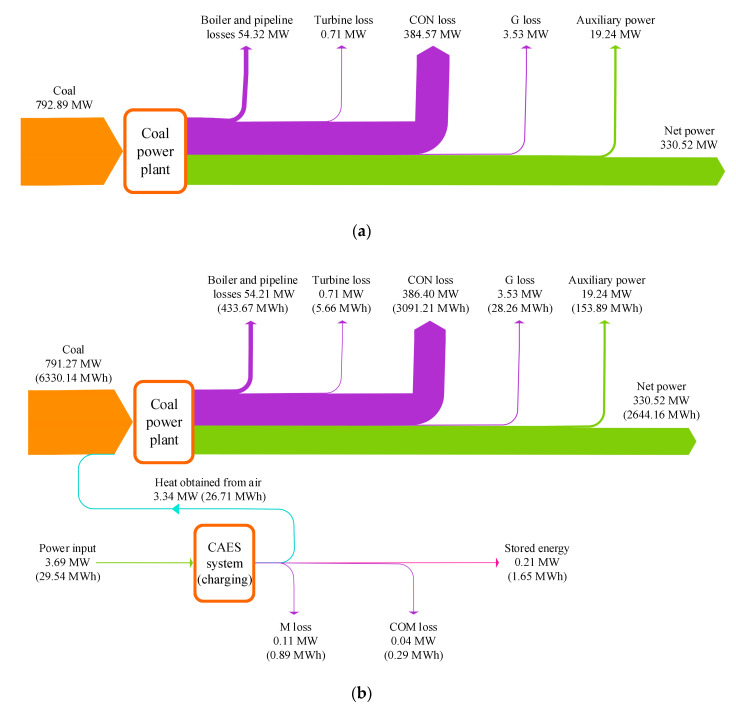

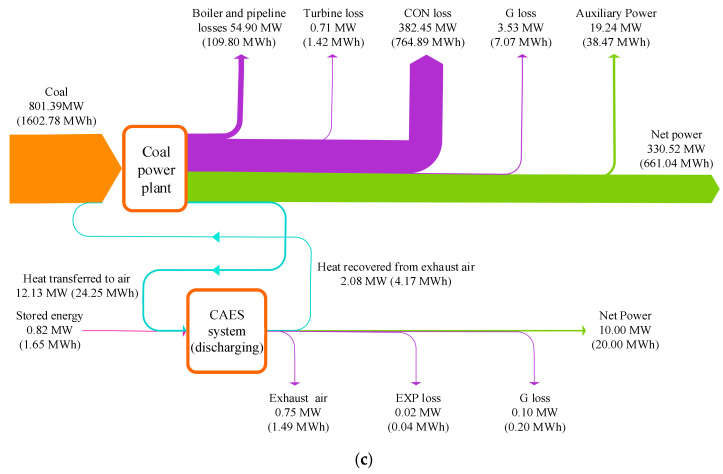
Energy flow diagrams of the reference coal power plant and proposed CAES system, (**a**) reference coal power plant, (**b**) charging process of the proposed CAES system, (**c**) discharging process of the proposed CAES system.

The detailed energy transfers and conversions that occurred in studied systems were examined and depicted in Figure 4. The fuel energy and the surplus electricity from the grid are recognized as the energy inputs of the hybrid system, and the net power of the coal power plant (330.52 MW) stays identical after the integration. The energy losses of the main components in the coal power section have no obvious changes. During the charging process of the CAES system, the feedwater heating system of the coal power plant absorbs 3.34 MW energy from the air, thereby the fuel energy input can be cut down by 1.62 MW. In the discharging period, the coal power plant conveys 12.13 MW heat energy to the CAES section, and the heat of 2.08 MW is recovered from the EXP2 outlet to the coal power plant. Regarding the entire energy storage process, 6.63 MWh energy is delivered from the CAES section to the coal power section, and the fuel energy input is increased by 4.02 MWh (as compared to the reference coal power in 10 h). Furthermore, the proposed CAES system can store 29.54 MWh electricity in off-peak hours and contribute to 20.00 MWh electricity when the power demand is strong.

### 4.4. Exergy Analysis

The exergy efficiency of the CAES system ($\eta_{\mathrm{ex},\mathrm{CAES}}$) has been defined to evaluate the proposed CAES system from the perspective of the second thermodynamic law, which means the ratio of the exergy output to the exergy input of the CAES system during the whole energy storage process:

(11) $$\eta_{\mathrm{ex},\mathrm{CAES}}=\frac{{EX}_{\mathrm{out},\mathrm{CAES}}}{{EX}_{\mathrm{in},\mathrm{CAES}}}$$

where $EX_{\mathrm{in},\mathrm{CAES}}$ is the exergy input of the CAES system, kW; $EX_{\mathrm{out}}$ is the exergy output of the CAES system, kW.

The exergy of a certain stream ($EX_{x}$, kW), such as air, steam, and water, can be calculated as:

(12) $$EX_{x}=m_{x}\times \left[\left(h_{x}-h_{x,0}\right)-T_{0}\times (s_{x}-s_{x,0})\right]$$

where $m_{x}$ is the flow rate of the stream, kg/s; $h_{x}$ and $h_{x,0}$ are the specific enthalpies of the stream at the present state and environmental state, kJ/kg; $s_{x}$ and $s_{x,0}$ are the specific entropies of the stream at the present state and environmental state, kJ/kgK; $T_{0}$ is the temperature of the environmental state, K.

**Figure 5:**
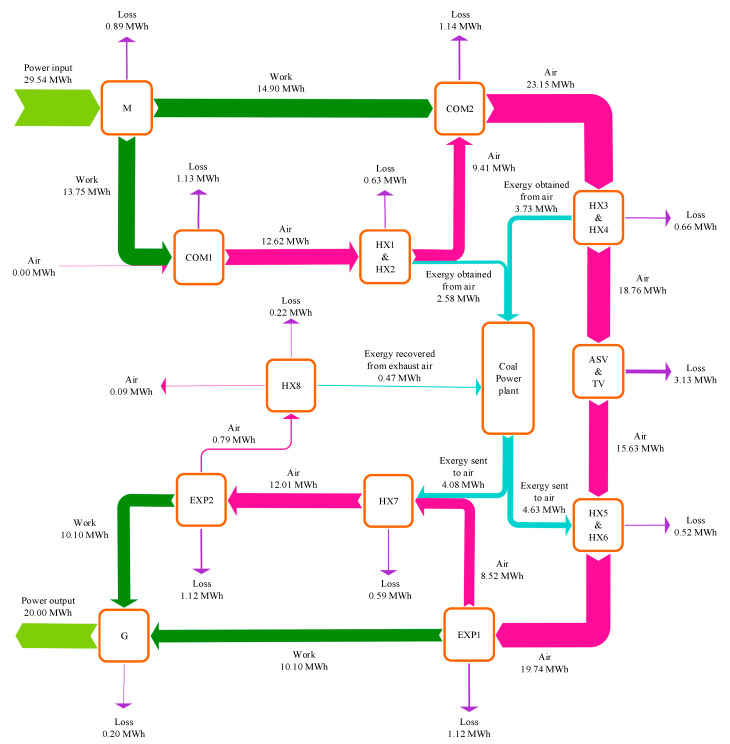
Exergy flow diagram of the proposed CAES system.

Figure 5 displays the exergy flow diagram of the hybrid CAES system containing the charging and discharging processes, which indicates the exergy inputs, exergy outputs, and exergy losses of the main equipment. During the charging period, the coal power plant absorbs 6.31 MWh exergy from the compressed air out of the COMs, and then 8.71 MWh exergy is transferred from the coal power plant to the air into the EXPs when discharging. The exhaust air of the EXP2 is exploited to heat a portion of the feedwater, and 0.47 MWh exergy can be avoided from wasting. The exergy of surplus electricity (29.54 MWh) is fed into the CAES system, and the exergy of 18.77 MWh is stored in the ASV via the compressed air. The overall exergy destruction in the ASV and the throttle valve (TV) is 3.13 MWh, which is the largest part of exergy loss in the CAES system. The energy storage and energy release are achieved through the COMs and the EXPs, where the exergy losses are 2.27 MWh and 2.24 MWh, respectively. The HX1-4 conduce to the recovery of the compression heat and improving the efficiency of the COM2, and HX5-7 are employed to warm the air from the ASV before the EXPs. The exergy destructions in the HX1-4 and HX5-8 are 1.29 MWh and 1.33 MWh. Besides, the total exergy recovered from the compressed air to the coal power section is less than the exergy delivered from the coal power plant to the compressed air, thereby the entire coal consumption rate is raised after the incorporation. Generally, the sum of the exergy flows into the CAES system is 26.78 MWh and the global exergy flowing out of the system is 18.25 MWh, as a consequence, the exergy efficiency of the CAES system can attain 70.01%.

## 5. Sensitivity Analysis

### 5.1. Effect of Ambient Temperature

**Figure 6:**
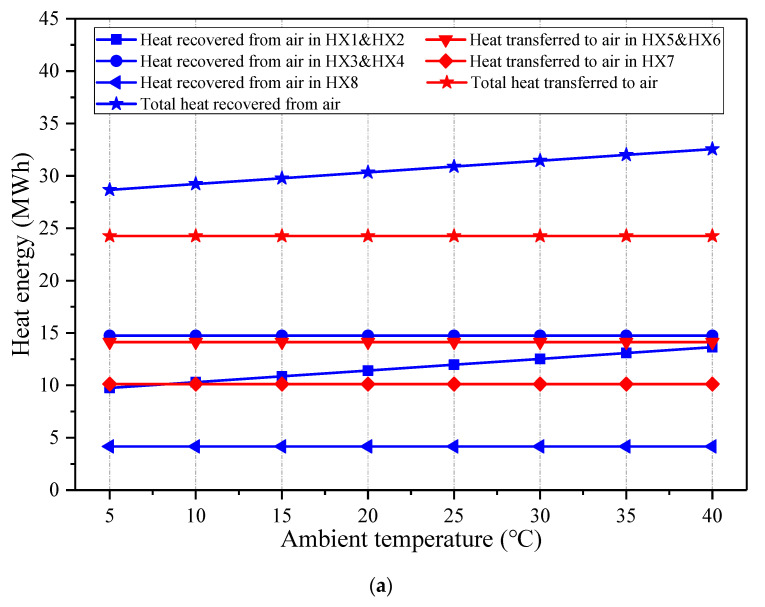

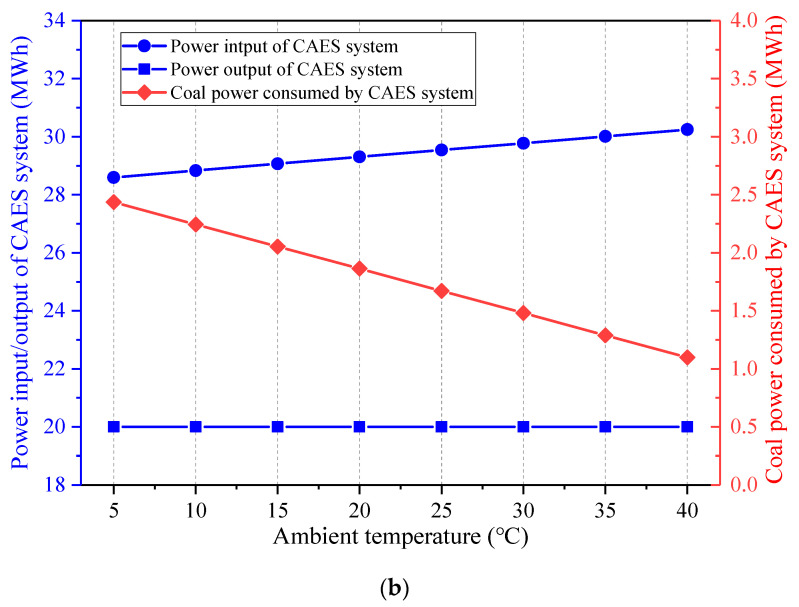
Influence of the ambient temperature on the main energy streams of the proposed CAES system, (**a**) heat energy recovered from and transferred to the air, (**b**) power streams and coal power consumed by the CAES system.

**Figure 7:**
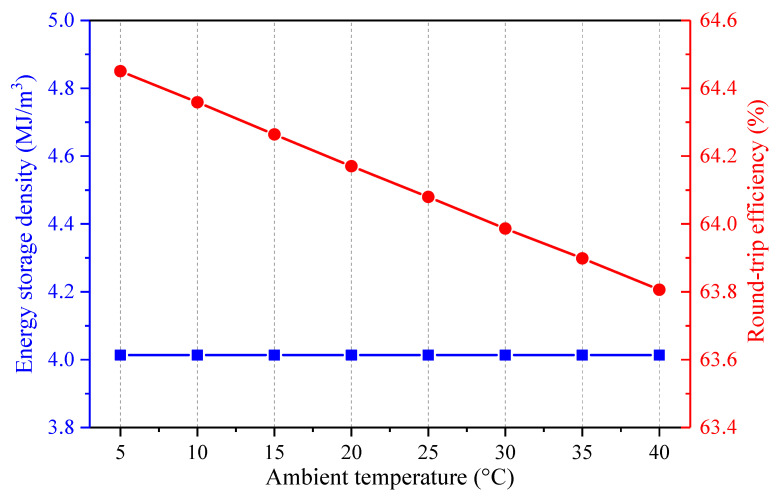
Influence of the ambient temperature on the performance of the proposed CAES system.

The impact of the ambient temperature on the energy flows of the proposed CAES system is illustrated in Figure 6. The variation of the ambient temperature mainly affects the air compression process, whereas, the air expansion process nearly remains constant. While the ambient air temperature grows, the heat recovered from the air in the HX1-2 and the total heat recovered from the air augment, but the other energy streams are inviable. The increment of the heat recovered from the air leads to the reduction of the coal power consumed by the CAES system. Furthermore, the power input of the CAES system increases, but the power production of the CAES system almost stays unchanged. As the power input of the CAES system is more sensitive than the coal power consumed by the CAES system with the change of the ambient temperatures, the round-trip efficiency of the CAES system declines with the decrement of the ambient air temperature, as shown in Figure 7. Since the volume of the ASV is maintained fixed, the energy storage density of the CAES system is unaltered. In general, a lower ambient temperature is more beneficial to the operation of the hybrid CAES system.

### 5.2. Effect of ASV Storage Pressure

**Figure 8:**
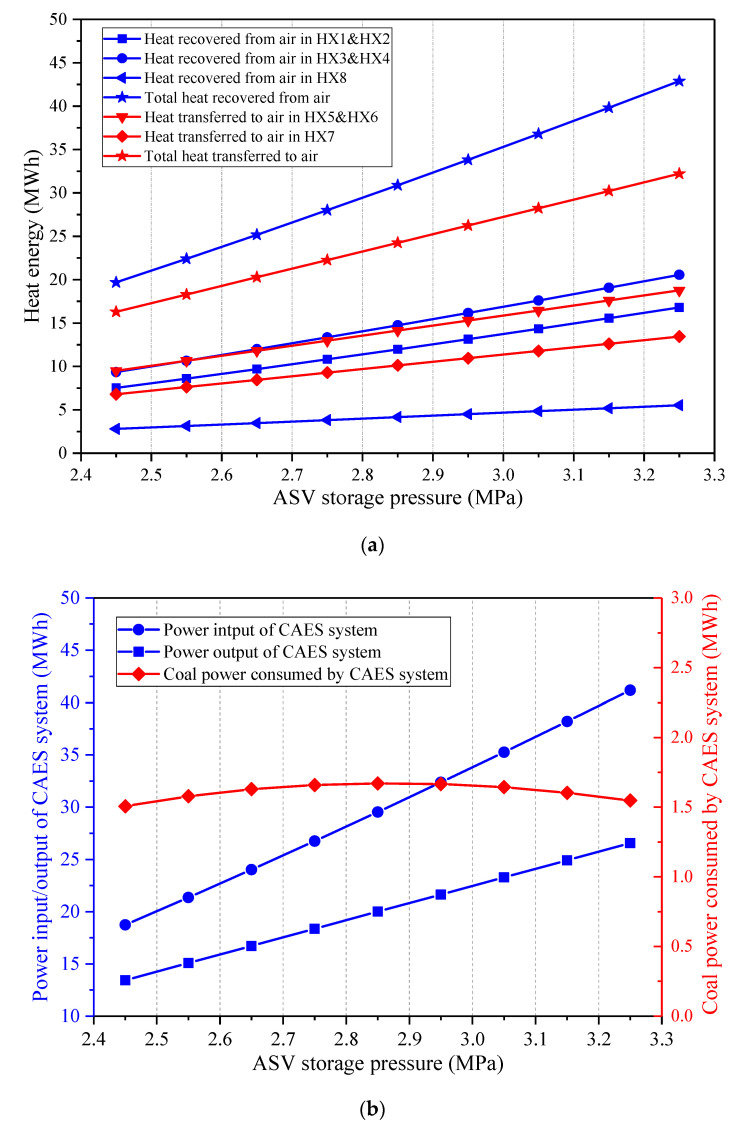
Influence of the ASV storage pressure on the main energy streams of the proposed CAES system, (**a**) heat energy recovered from and transferred to the air, (**b**) power streams and coal power consumed by the CAES system.

**Figure 9:**
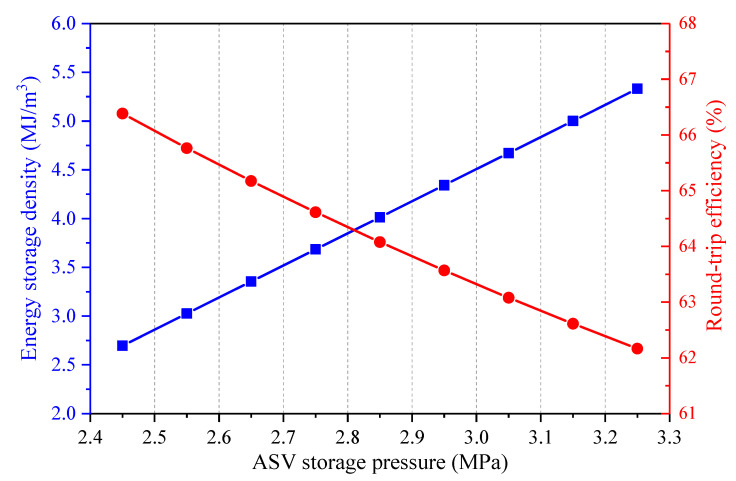
Influence of the ASV storage pressure on the performance of the proposed CAES system.

Figure 8 presents how the ASV storage pressure affects the primary energy flows in the CAES system. The charging process and discharging process of the CAES system are both impacted by the ASV storage pressure. The volume of the ASV is considered unchanged under various storage pressures, thereby the increment of the storage pressure leads to the growths of the compression ratios of the COMs, and the total mass amount of the stored air rises. Consequently, the heat amounts obtained from and transferred to the air increase with the augment of the ASV storage pressure. As the power consumption of the CAES system’s motor and the power generation of the CAES system’s generator are regarded as fixed, the charging time and the discharge time of the CAES system are extended. Hence, both the power input and power output of the CAES system are raised. What’s more, the inlet pressure of the EXP1 is throttled to keep a constant value, thus, the trend of the power input of the CAES system is steeper than that of the power output of the CAES system. Further, the coal power consumed by the CAES system changes little under different storage pressures. As a consequence, the round-trip efficiency of the CAES system dwindles with the increment of the storage pressure, as displayed in Figure 9. Since more air can be stored in the ASV when the storage pressure is larger, the energy storage density will be improved.

### 5.3. Effect of EXP1 Inlet Temperature

**Figure 10:**
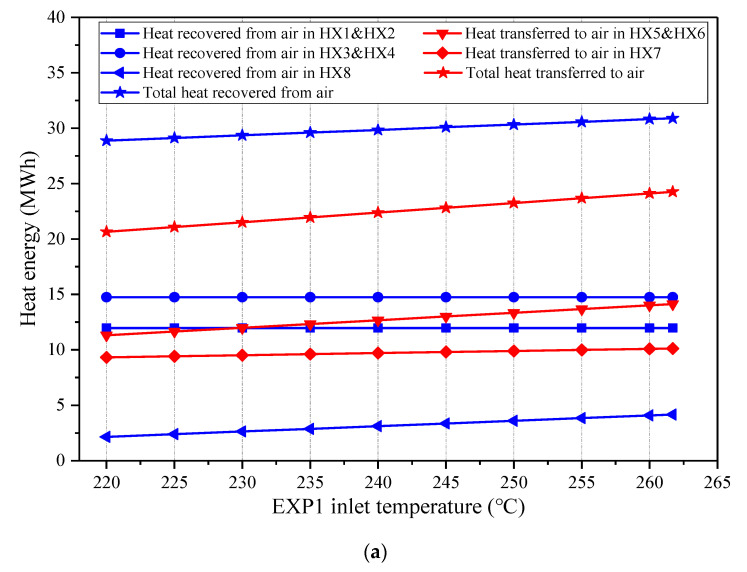

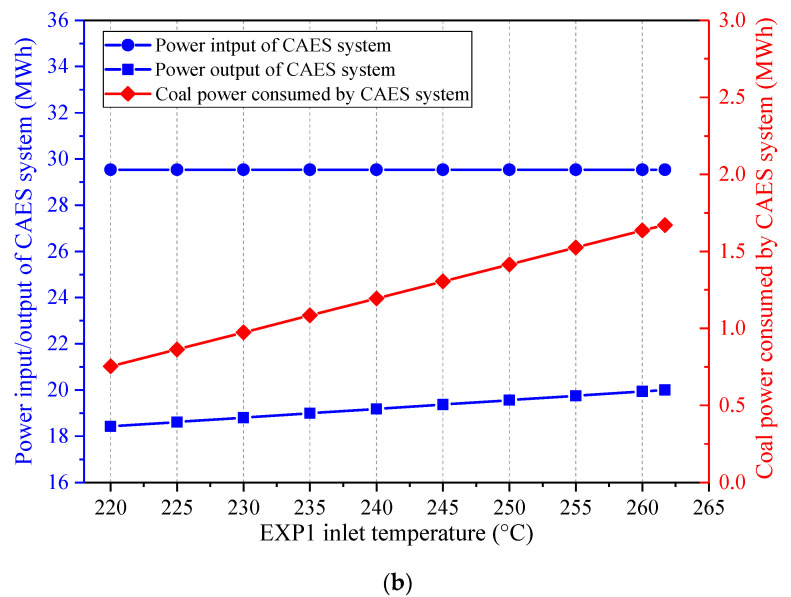
Influence of the EXP1 inlet temperature on the main energy streams of the proposed CAES system, (**a**) heat energy recovered from and transferred to the air, (**b**) power streams and coal power consumed by the CAES system.

**Figure 11:**
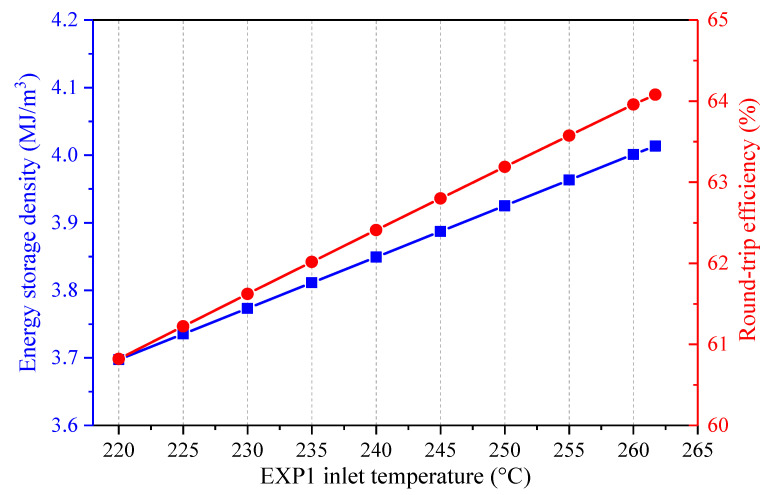
Influence of the expander 1 (EXP1) inlet temperature on the performance of the proposed CAES system.

The influence of the EXP1 inlet temperature on the main energy streams of the CAES system is illustrated in Figure 10. The inlet air temperature of the EXP2 is maintained identical to that of the EXP1. The variation of the EXP1 inlet temperature only affects the discharging process of the CAES system, therefore the total mass amount of the stored air and the heat amounts recovered in the HX1-4 remain invariable. Once the EXP1 inlet temperature increases, more heat energy from the feedwater is required to heat the air into the EXPs and the heat exchange capacities of the HX5-8 augment. Hence, the coal power consumed by the CAES system rises. With the increasing EXP1 inlet temperature, the specific power generation capacity of the discharging air increases, resulting in the decrement of the air flow rate in the EXPs and a longer discharging duration. Hence, the energy storage density of the CAES system can be enhanced with a higher EXP1 inlet temperature, as indicated in Figure 11. Meanwhile, the round-trip efficiency of the CAES will be promoted.

### 5.4. Effect of Coal Power Plant Load

**Figure 12:**
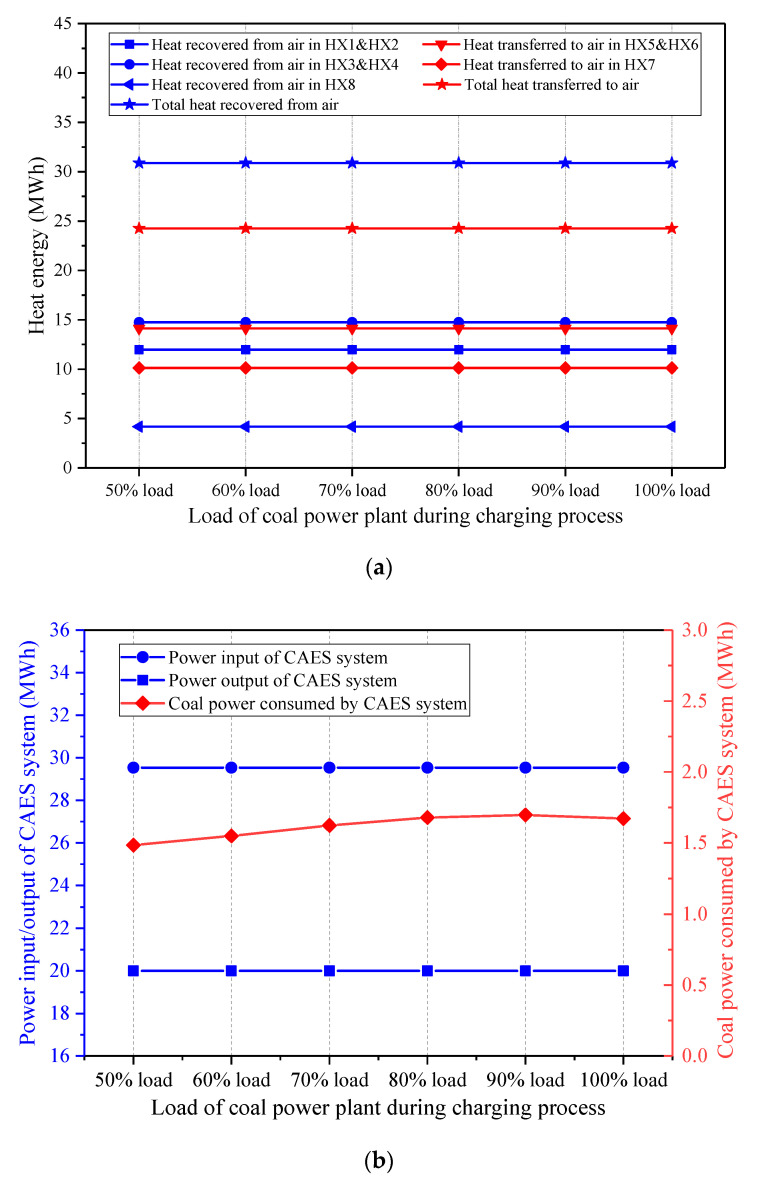
Influence of the coal power plant load during the charging process on the main energy streams of the proposed CAES system, (**a**) heat energy recovered from and transferred to the air, (**b**) power streams and coal power consumed by the CAES system.

**Figure 13:**
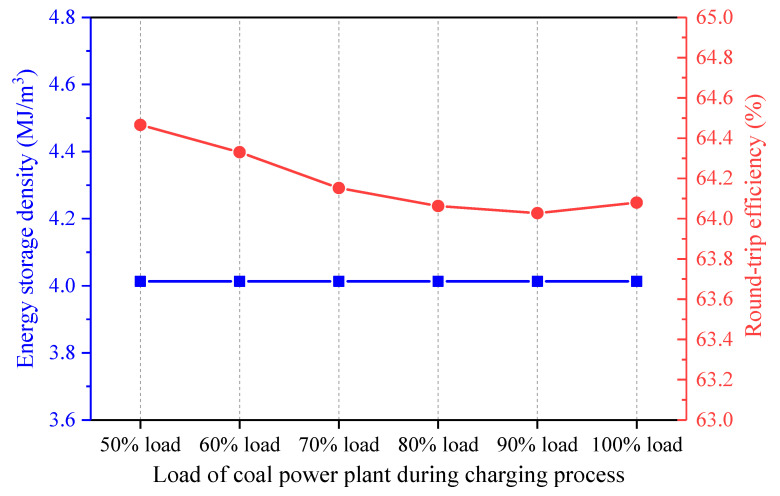
Influence of the coal power plant load during the charging process on the performance of the proposed CAES system.

The operation of the coal power plant is closely related to that of the CAES system in the integrated design. The effect of the coal power plant load on the performance of the CAES system was investigated. The coal-to-electricity efficiency may change under various loads of the coal power plant, but the coal-to-electricity efficiency and the coal power output are maintained constant for the single coal power plant and the hybrid system. Thus, the coal amount used for power generation by the coal power plant is regarded as identical for the single coal power plant and the hybrid power system. Figure 12 shows the influence of the coal power plant load during the charging process on the energy streams of the hybrid system, while the coal power plant load keeps constant in the discharging process. While the coal power load during the charging process varies from 50 to 100%, the other parameters of the proposed system (e.g., the coal power plant load during the discharging process, the charging/discharging process parameters of the CAES system, etc.) are maintained constant. Therefore, the change of the coal power load during the charging process has no effect on the charging process of the CAES system, and the total heat recovered from the air during the charging period remains unchanged. Since the coal power load during the discharging process stays fixed, the coal power plant operation does not affect the discharging process of the CAES system. Above all, the heat amounts obtained or delivered to the air and the power inputs/outputs remain invariable when the coal power plant load during the charging process is boosted. Further, the coal power used by the CAES system does not change much. Consequently, the energy storage density of the CAES system remains constant, while the round-trip efficiency of the CAES system declines slightly, as displayed in Figure 13.

**Figure 14:**
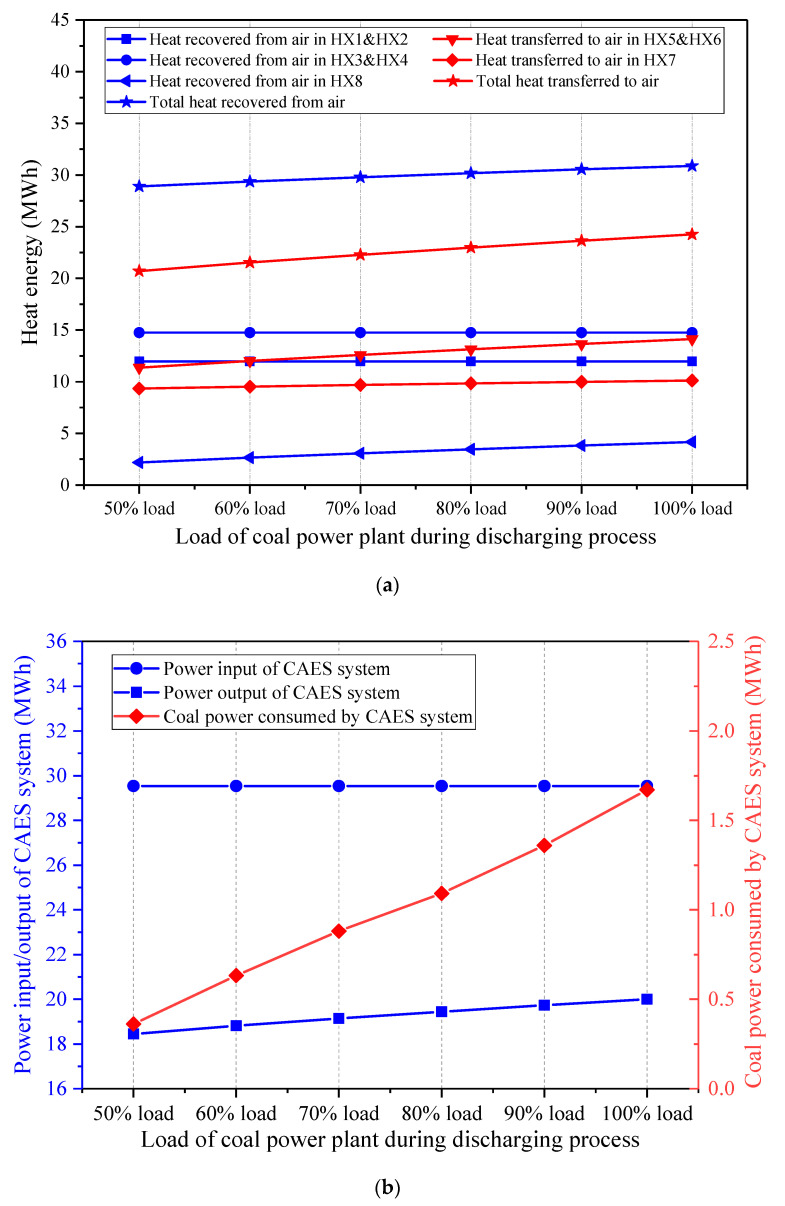
Influence of the coal power plant load during the discharging process on the main energy streams of the CAES system, (**a**) heat energy recovered from and transferred to the air, (**b**) power streams and coal power consumed by the CAES system.

**Figure 15:**
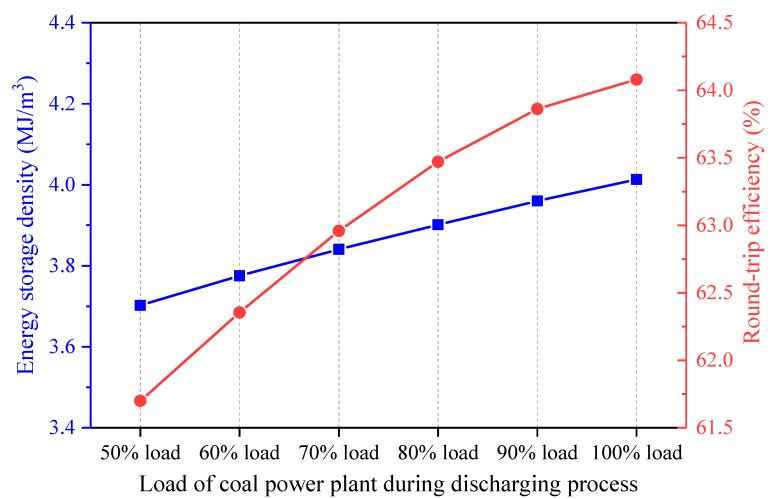
Influence of the coal power plant load during the discharging process on the performance of the proposed CAES system.

Once the coal power plant load during the charging process remains unchanged, and the coal power plant load during the discharging process varies, the effect on the energy streams of the hybrid CAES system is presented in Figure 14. The change of the coal power load during the discharging process impacts the expansion side of the CAES system, whereas the compression side and the total mass of the stored air are impervious. Hence, the heat exchange capacities in the HX5-8 rise with the increment of the coal power plant load during the discharging process, but the heat amounts recovered from the air during the charging process stays the same. The energy streams and the performance of the CAES system under different coal power loads during the discharging process have similar trends with those of the CAES system affected by the EXP1 inlet temperatures. If the coal power plant load during the discharging process becomes larger, both the power output of the CAES system and the coal power consumed by the CAES system will be enhanced. Furthermore, Figure 15 indicates that a higher coal power plant load during the discharging process contributes to a larger energy storage density and round-trip efficiency of the CAES system, which is conducive to the operation of the hybrid CAES system.

## 6. Further Discussion

The novel CAES system is designed to store the surplus electricity of the grid during the off-peak hours and release the stored energy once the electric demand gets high. As compared to a typical CAES system, the thermal energy storage equipment is not needed because of the incorporation, while eight HXs are exploited to recover/transfer heat between the CAES system and the coal power plant. The capital cost of the thermal energy storage subsystem accounts for nearly 12.75% of the total capital cost of a CAES system [40]. For the study case of this research, 851.89 thousand USD is estimated to be saved due to the removal of the thermal energy storage equipment [40,41]. Moreover, the total capital cost of the HXs is also brought down by 1532.92 thousand USD, although the number of the HXs increases. Generally, the total capital cost of the CAES system can be reduced by 2384.81 thousand USD when adopting the proposed design, namely, the total capital cost of the CAES system declines by 35.69% in contrast to the conventional CAES system. Therefore, the new CAES system has the significant advantage of low investment and will be more profitable than a traditional CAES system. The profit of a CAES system is highly dependent on the electricity market, and a further economic investigation will be conducted in our future study.

## 7. Conclusions

To improve the performance of a CAES system, an innovative design that integrates a CAES system into a coal power plant was put forward. In the hybrid scheme, partial feedwater of the coal power plant absorbs the compression heat from the CAES section during the charging process, while the compressed air prior to the expanders is warmed by the feedwater taken from the coal power plant during the discharging process. Besides, the waste energy of the exhaust air of the EXP 2 is recovered by the feedwater as well. Through the coupling of the CAES system and coal power plant, the thermal energy storage equipment for a typical CAES system is unnecessary, and energy cascade utilization can be achieved, thereby the performance of the CAES system is enhanced. Based on a 350 MW coal power plant, the novel concept was evaluated from the perspectives of energy and exergy. Once the power production of the coal power plant is fixed, the round-trip efficiency of the new CAES system can attain 64.08%, with an energy storage density of 4.01 MJ/m^3^. The most significant exergy loss comes from the ASV and TV, followed by the compressors and the expanders, and the exergy efficiency of the CAES system is 70.01%. A sensitivity analysis was conducted to investigate the performance of the proposed CAES system under various conditions. The increments of the ambient temperature and air storage pressure cause negative effects on the performance of the CAES system, whereas, an increase of the EXP 1 inlet temperature results in an improvement in the round-trip efficiency. Moreover, the change of the coal power plant load during the charging process has little influence on the operation of the CAES system, but the impact of the coal power plant load during the discharging process has similar trends with that of the EXP 1 inlet temperature. Via the suggested integration, the total capital cost of the CAES system can be reduced by 35.69%. Above all, the novel concept is highly suitable and favorable from the thermodynamic and economic aspects.

## Figures and Tables

**Table 1:** Basic parameters of the reference coal-fired power plant.

Item		Unit	Value
Coal consumption rate		kg/s	42.29
Net caloric value of coal		kJ/kg	18,750
Main steam (into turbine)	Pressure	MPa	24.20
	Temperature	°C	566.0
	Flow rate	kg/s	274.80
Reheated steam (into turbine)	Pressure	MPa	3.78
	Temperature	°C	566.0
	Flow rate	kg/s	232.33
Exhaust steam (out of turbine)	Pressure	kPa	4.90
	Temperature	°C	32.5
	Flow rate	kg/s	174.31
Gross power		MW	349.76
Net power		MW	330.52
Coal-to-electricity efficiency		%	41.69

**Table 2:** Model validation based on the reference coal-fired power plant (100% load).

Item		Design	Simulation	Relative Error (%)
Coal consumption rate (kg/s)		42.29	42.29	0.00
Main steam (into turbine)	Pressure (MPa)	24.2	24.2	0.00
	Temperature (°C)	566.0	566.0	0.00
	Flow rate (kg/s)	274.80	274.80	0.00
Reheated steam (into turbine)	Pressure (MPa)	3.78	3.78	0.00
	Temperature (°C)	566.0	566.0	0.00
	Flow rate (kg/s)	232.33	232.36	+0.01
Exhaust steam (out of turbine)	Pressure (kPa)	4.90	4.90	0.00
	Temperature (°C)	32.5	32.5	0.00
	Flow rate (kg/s)	174.31	174.29	−0.01
Feedwater (into boiler)	Pressure (MPa)	26.23	26.23	0.00
	Temperature (°C)	276.4	276.4	0.00
	Flow rate (kg/s)	274.80	274.80	0.00
Exhaust gas temperature (°C)		130.0	130.0	0.00
Boiler efficiency (%)		94.09	94.09	0.00
Gross power (MW)		349.76	350.00	+0.07
Net power (MW)		330.52	330.75	+0.07
Coal-to-electricity efficiency (%)		41.69	41.72	+0.07

**Table 3:** Model validation based on the CAES system in Ref. [37].

Item	Ref. [37]	Simulation	Relative Error (%)
Power consumption of COM1 (kW)	485.57	486.00	+0.09
Work consumption of COM2 (kW)	514.43	514.63	+0.04
Total power consumption during charging process (kW)	1000	1000.63	+0.06
Air flow rate during charging process (kg/s)	1.58	1.58	0.00
Work generation of EXP1 (kW)	509.37	509.65	+0.05
Work generation of EXP2 (kW)	490.63	491.11	+0.10
Total power generation during discharging process (kW)	1000	1000.76	+0.08
Air flow rate during discharging process (kg/s)	2.36	2.36	0.00
Charge time (h)	6.06	6.06	0.00
Discharge time (h)	4.06	4.06	0.00
Round-trip efficiency (%)	66.98	67.01	+0.04

**Table 4:** Basic parameters of the proposed CAES system [35,38,39].

Item		Unit	Value
ASV	Volume	m^3^	17,940
	Air temperature	°C	50.0
	Storage pressure	MPa	2.85
	Release pressure	MPa	1.63
Isentropic efficiency of COM		%	88
Isentropic efficiency of EXP		%	88
Charging time		h	8
Discharging time		h	2

**Table 5:** Energy performance of the proposed CAES system.

Item	Reference Coal Power Plant	Proposed System	Variation
Charging process			
Load of coal power plant	100% Load	100% Load	-
Net power of coal power plant (MW)	330.52	330.52	0
Coal consumption rate of coal power plant (kg/s)	42.29	42.20	−0.09
Coal power conserved by CAES system (MWh)	-	5.41	-
Power consumption of CAES system’s motor (MW)	-	3.69	-
Power input of CAES system during charging (MWh)	-	29.54	-
Discharging process			
Load of coal power plant	100% Load	100% Load	-
Net power of coal power plant (MW)	330.52	330.52	0
Coal consumption rate of coal power plant (kg/s)	42.29	42.74	+0.45
Coal power consumed by CAES system (MWh)	-	7.08	-
Power generation of CAES system’s generator (MW)	-	10.00	-
Power output of CAES system during discharging (MWh)	-	20.00	-
Performance indicators			
Round-trip efficiency of CAES system (%)	-	64.08	-
Energy storage density of CAES system (MJ/m^3^)	-	4.01	-
Total coal consumption variation in charging and discharging process (kg)	-	769.59	-
Total coal power consumed by CAES system (MWh)	-	1.67	-
Overall efficiency of hybrid system (%)	-	41.76	-

## Nomenclature

**Symbols**			
*EX*	exergy (kW)	*s*	specific entropy (kJ/kgK)
*h*	specific enthalpy (kJ/kg)	*t*	time (h)
*m*	flow rate (kg/s)	*T*	temperature (K)
*P*	power (kW)	*V*	volume (m^3^)
*Q*	energy (kWh)	*W*	work (kWh)
*q*	caloric value (kJ/kg)	*η*	efficiency
**Subscripts**			
0	environmental state	hyb	hybrid
c	coal	in	inlet
ch	charge	out	outlet
c-e	coal-to-electricity	ref	reference
disch	discharge	x	certain stream
ex	exergy		
**Abbreviations**			
ASV	air storage vessel	G	generator
CAES	compressed air energy storage	HPT	high-pressure turbine
COM	compressor	HX	heat exchanger
CON	condenser	IPT	intermediate-pressure turbine
CP	condensate pump	LPT	low-pressure turbine
DEA	deaerator	M	motor
ESD	energy storage density (kJ/m^3^)	RH	regenerative heater
EP	extra pump	RTE	round-trip efficiency (%)
EXP	expander	TV	throttle valve
FWP	feedwater pump		
